# Effects of a Gamified Agent-Based System for Personalized Elderly Care: Pilot Usability Study

**DOI:** 10.2196/48063

**Published:** 2023-11-23

**Authors:** Diogo Martinho, Vítor Crista, João Carneiro, Kenji Matsui, Juan Manuel Corchado, Goreti Marreiros

**Affiliations:** 1 Research Group on Intelligent Engineering and Computing for Advanced Innovation and Development Polytechnic of Porto - School of Engineering (ISEP) Porto Portugal; 2 Osaka Institute of Technology Osaka Japan; 3 Grupo de investigación en Bioinformática, Sistemas Informáticos Inteligentes y Tecnología Educativa Salamanca Spain

**Keywords:** gamification, cognitive assistants, elderly care, coaching system, older people, technology, virtual assistant, cognitive, usability, intervention, physical activity, agent-based system

## Abstract

**Background:**

The global percentage of older people has increased significantly over the last decades. Information and communication technologies have become essential to develop and motivate them to pursue healthier ways of living. This paper examines a personalized coaching health care service designed to maintain living conditions and active aging among older people. Among the technologies the service includes, we highlight the use of both gamification and cognitive assistant technologies designed to support older people and an application combining a cognitive virtual assistant to directly interact with the older person and provide feedback on their current health condition and several gamification techniques to motivate the older person to stay engaged with the application and pursuit of healthier daily habits.

**Objective:**

This pilot study aimed to investigate the feasibility and usability of a gamified agent-based system for older people and obtain preliminary results on the effectiveness of the intervention regarding physical activity health outcomes.

**Methods:**

The study was designed as an intervention study comparing pre- and posttest results. The proposed gamified agent-based system was used by 12 participants over 7 days (1 week), and step count data were collected with access to the Google Fit application programming interface. Step count data after the intervention were compared with average step count data before the intervention (average daily values over a period of 4 weeks before the intervention). A　1-tailed Student *t* test was used to determine the relationship between the dependent and independent variables. Usability was measured using the System Usability Scale questionnaire, which was answered by 8 of the 12 participants in the study.

**Results:**

The posttest results showed significant pre- to posttest changes (*P*=.30; 1-tailed Student *t* test) with a moderate effect size (Cohen *d*=0.65). The application obtained an average usability score of 78.

**Conclusions:**

The presented pilot was validated, showing the positive health effects of using gamification techniques and a virtual cognitive assistant. Additionally, usability metrics considered for this study confirmed high adherence and interest from most participants in the pilot.

## Introduction

We are currently witnessing an increase in the world’s population, which is also growing older. According to United Nations’ World Population Ageing Report [1], the global population aged 65 years or older was 727 million in 2020 and is expected to reach over 1.5 billion in 2050. With the increase in the number of older citizens living in today’s society, there has been a growing necessity to research and develop assistive technologies that can support older people [2,3], especially in intelligent environments [4,5]. Among the already available technologies, we highlight the use of gamification and cognitive assistants in this context.

Gamification refers to enhancing services with features that can offer “gameful” experiences to their users and keep them motivated and engaged, increasing their activity, social interaction, and the quality and productivity of their actions [6]. In other words, gamification can be seen as improving applications developed for serious contexts using game-like features that can potentiate user experience and motivate and encourage the user to pursue desired behaviors.

Companies are extending (and gamifying) their existing services, and more investments are being made in developing gamified applications and serious games [6]. Likewise, recent research has led to advancements in gamification and serious games in health care [7-10] with great focus on different health domains, such as promoting both physical and cognitive activity [11-23], promoting behavioral changes [7,24] and providing personalized health care services at all ages [25]. These services can bring innovative and cost-effective solutions and treatments to the most fragile and isolated groups in our society, such as older people and those with chronic diseases [14,26]. Furthermore, personalization becomes essential for these kinds of services, as individual necessities and capabilities are even more impactful to this age group. The lack of personalized mechanisms could lead to quick loss of interest in using the health care service. In elderly care, this could easily result in the deterioration of health conditions.

Cognitive assistant research and development has seen significant advances in the literature over the last decade. Cognitive assistants can be endowed with social and emotional processing [27,28] to interact with humans and improve their capabilities while not replacing them in specific tasks. In a more formal definition, cognitive assistants can be described as intelligent mechanisms that interact and learn with the user and can provide adequate feedback to improve behaviors that otherwise would be difficult to accomplish. As such, cognitive assistants will augment human intelligence and assist in decision-making and action-taking [29]. In recent years, exciting approaches have been proposed targeted toward elderly care to enhance the daily lives of older people and improve both their cognitive and physical capabilities.

In sum, the development of intelligent mechanisms and algorithms to adjust the interaction and support provided to the older person, including serious games with a scalable difficulty as well as robots that play games and communicate with older people using different emotions, is fundamental to ensure interactive and personalized systems that are tailored to each user [30].

Existing research on combining the use of a gamified application with the use of a virtual cognitive assistant for the context of elderly care is still lacking. According to a previous study [31] and most recent proposals in the literature, the majority of the available approaches using some kind of assistant either consider the use of a physical robot or a virtual companion (usually a pet) to interact with the older person; neither approach considers the user’s progress as a variable in their design. As a result, these approaches cannot follow the evolution of the user over time and are most frequently used for one-time interactions. Because of that, they can represent user progress in adopting improved, healthier behaviors only in a very limited way. Therefore, the major novelty proposed in this work is a cognitive virtual assistant that can take advantage of the outcomes of a gamified health care application. Such an assistant can not only react emotionally to the user’s progress (in terms of achieved health goals and challenges) but also learn how to interact and improve the feedback provided to them by learning their behaviors, interests, and current capabilities. In this regard, we studied the use of processes to provide feedback and rewards to the older person, make it possible to increase the cognitive and physical load as the user becomes more proficient with the system, and adopt methods that enhance and promote social interaction, which can result in more personalized elderly care solutions. Furthermore, adapting the support provided to the older person according to their interests, capabilities, and necessities, as well as the surrounding environment, will improve health and well-being, capture interest and ensure positive engagement, facilitate social interaction, and decrease the impact of many medical conditions.

Considering all these ideas, this study aims to perform an initial validation to compare outcomes after using a health care application developed for older people named CoaFEld (Coaching for Elderly) and to determine its overall acceptability and usability after the experiment period is completed. For this, we chose user step count data as the primary outcome. We formulated a hypothesis through a paired 1-tailed Student *t* test to compare step count data obtained before and after the intervention.

## Methods

### CoaFEld System Design

**Figure 1:**
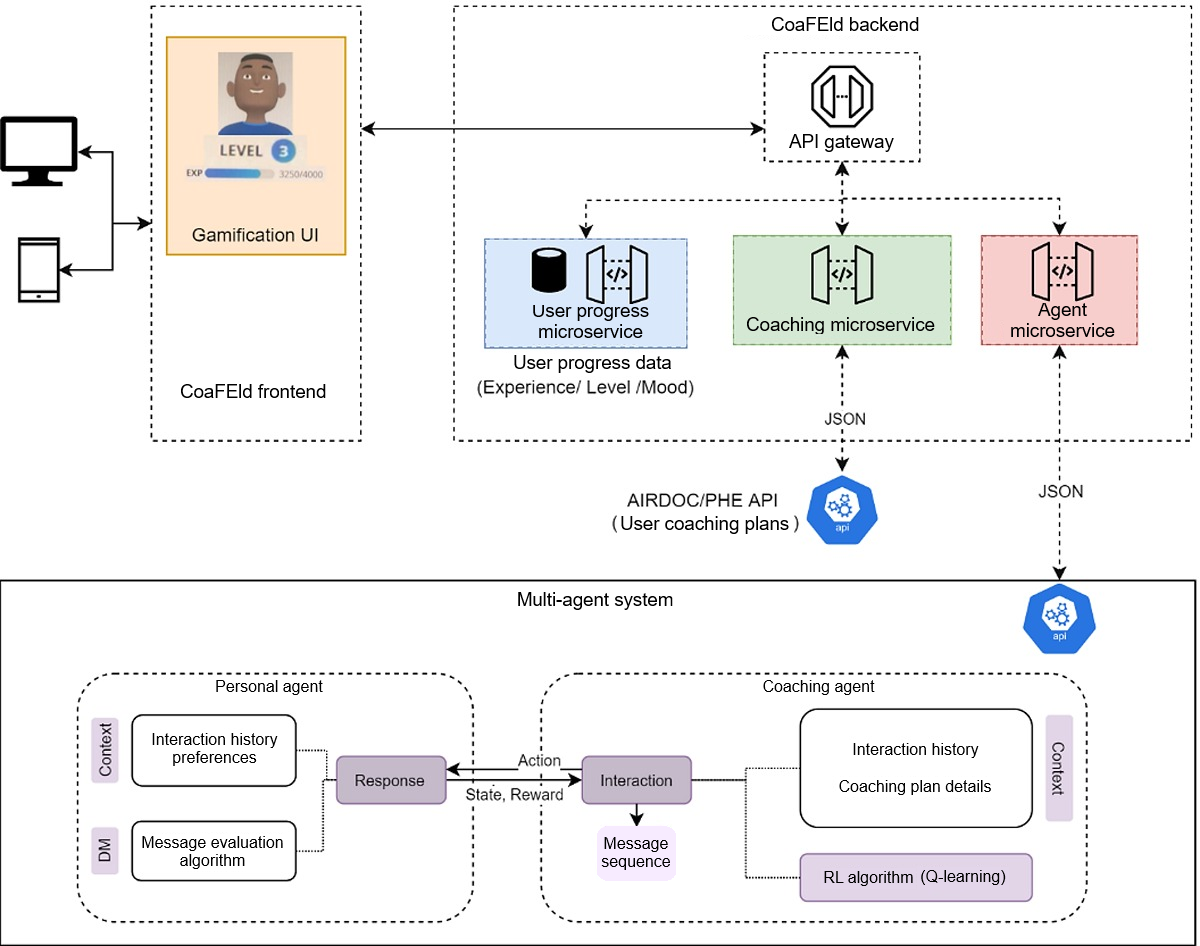
The proposed coaching system architecture reproduced from Martinho et al [30] which is published under Creative Commons Attribution 4.0 International License [32]. AIRDOC: Aplicação móvel inteligente para suporte individualizado e monitorização da função e sons respiratórios de doentes obstrutivos crónicos; API: application programming interface; CoaFEld: Coaching for Elderly; DM: decision making; RL: reinforcement learning; PHE: personal health empowerment; UI: user interface.

CoaFEld was developed following a microservice-oriented architecture (Figure 1), divided into 3 main components: a user web application, an application programming interface (API) gateway, and a set of microservices. These microservices communicate and store specific user information regarding user progress while using the application (using different gamification components), health status (derived by the performance of established coaching plans), and user interactions with the feedback that is provided to the user (personalized message interactions based on user preferences).

The coaching application that has been developed is gamified with consideration of the main findings of Martinho et al [31], who reported the most successful gamification techniques in the context of elderly care. These techniques include features such as experience or levels and points as the system’s currency to unlock new content. Additionally, the developed application presents a cognitive virtual assistant with different emotional queues depending on the positive or negative progress of older people as players and users of the coaching application. Following Martinho et al [30], the current emotional state shown by the cognitive assistant is based on all coaching plans assigned to that user and an evaluation of the number of goals that have been successfully achieved by that user vs the total number of goals in the coaching plan. The details of these plans are described in more detail in our previous work [33].

**Figure 2:**
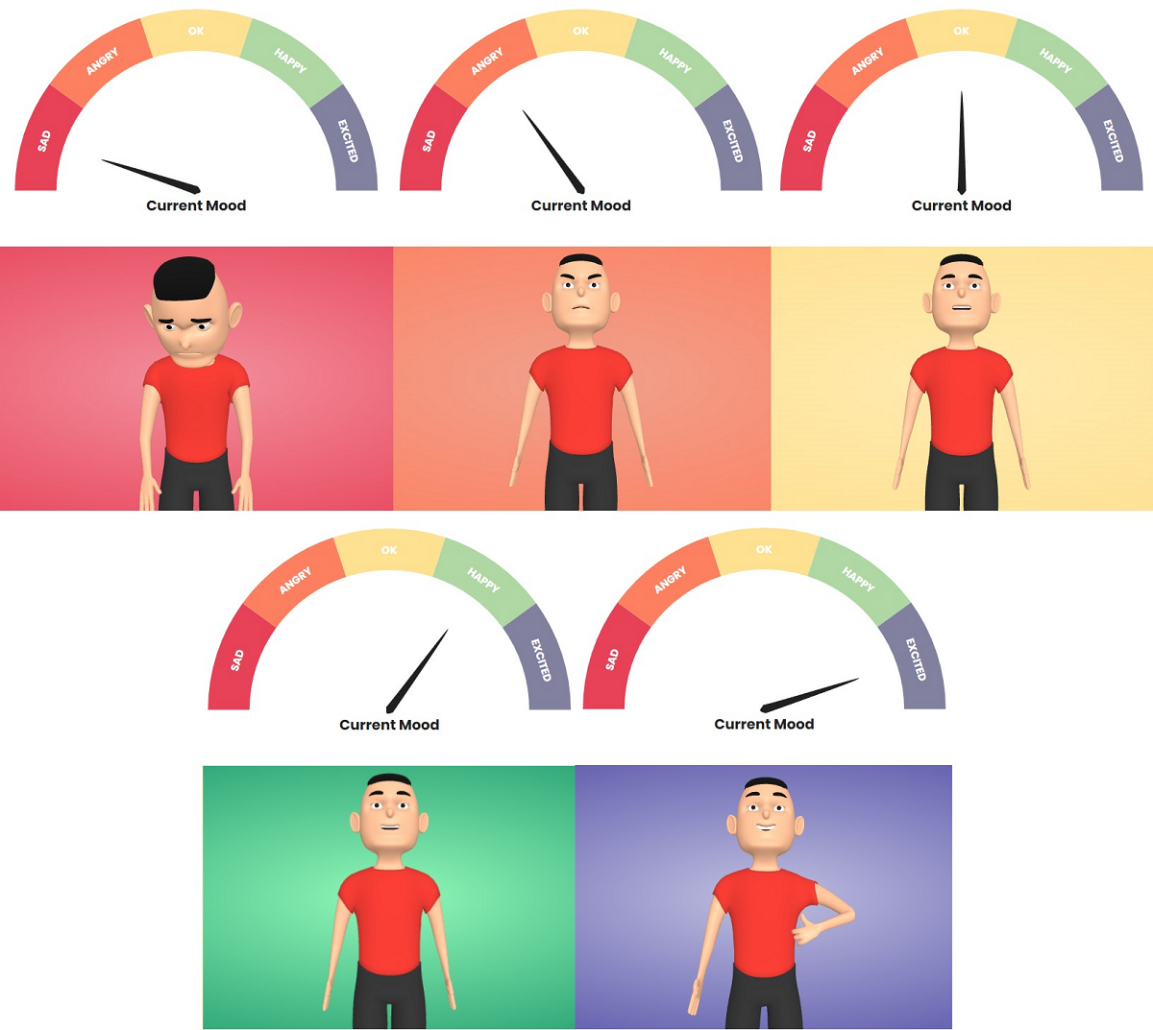
Representation of the 5 different emotional states of the cognitive assistant reproduced from Martinho et al [30] which is published under Creative Commons Attribution 4.0 International License [32].

We consider 5 main emotional states to represent user progress (Figure 2). These emotional states were inspired by Russell’s [34] circumplex model of affect, which is based on 2 dimensions (arousal and valence) and is ranked according to degree for both of these dimensions.

(1) The “sad” or “depressed” emotions are the most negative (with negative degrees for both arousal and valence); (2) “angry” represents the second most negative emotion (with a positive degree for arousal and a negative degree for valence); (3) the “OK” or “calm” emotions are moderate (with a negative degree for arousal and a positive degree for valence); (4) “happy” represents the second most positive emotion (with a neutral degree for arousal and a positive degree for valence); (5) “excited” represents the most positive emotion (with positive degrees for both arousal and valence).

An API gateway was developed in the proposed work with the main goal of encapsulating a set of several microservices and providing an API specific for each client (thus serving as a single entry point into the system). It is also the responsibility of the API gateway to include authorization and authentication functionalities and assure asynchronous communication between the requests sent to the available microservices. Three microservices have been developed to store and provide information related to both user progress and health outputs:

The “user progress” microservice manages data related to user progress and actions within the coaching application, including all the information on current game experience, level, and points; unlocked content; in-game purchases; cognitive play session results; and virtual assistant configurations and emotional state. The “coaching” microservice, developed by Martinho et al [33], is a generic mechanism to configure and evaluate coaching plans with health-related goals that can be dynamically updated with increasing or decreasing difficulty depending on the user’s performance. The “agent” microservice, developed by Martinho et al [35], uses a personal agent that returns the interaction (message queue) to be sent directly to the user and evaluates the feedback provided by the user and a coaching agent that applies reinforcement learning to understand what interactions should be next sent to the user at specific moments of the day based on previous interactions with that user.

### Mobile Interface Design

The CoaFEld application was developed to establish an environment where users can improve different aspects of their daily lives. In this preliminary validation, the study focused on accessing the user’s current physical condition, specifically daily step data, which is retrieved from the Google Fit API (after the user grants permission to access this information). After this, daily step challenges are proposed to the user based on the number of steps they have taken weekly. Multimedia Appendix 1 shows the algorithm used to combine the step data information retrieved from the Google API.

**Figure 3:**
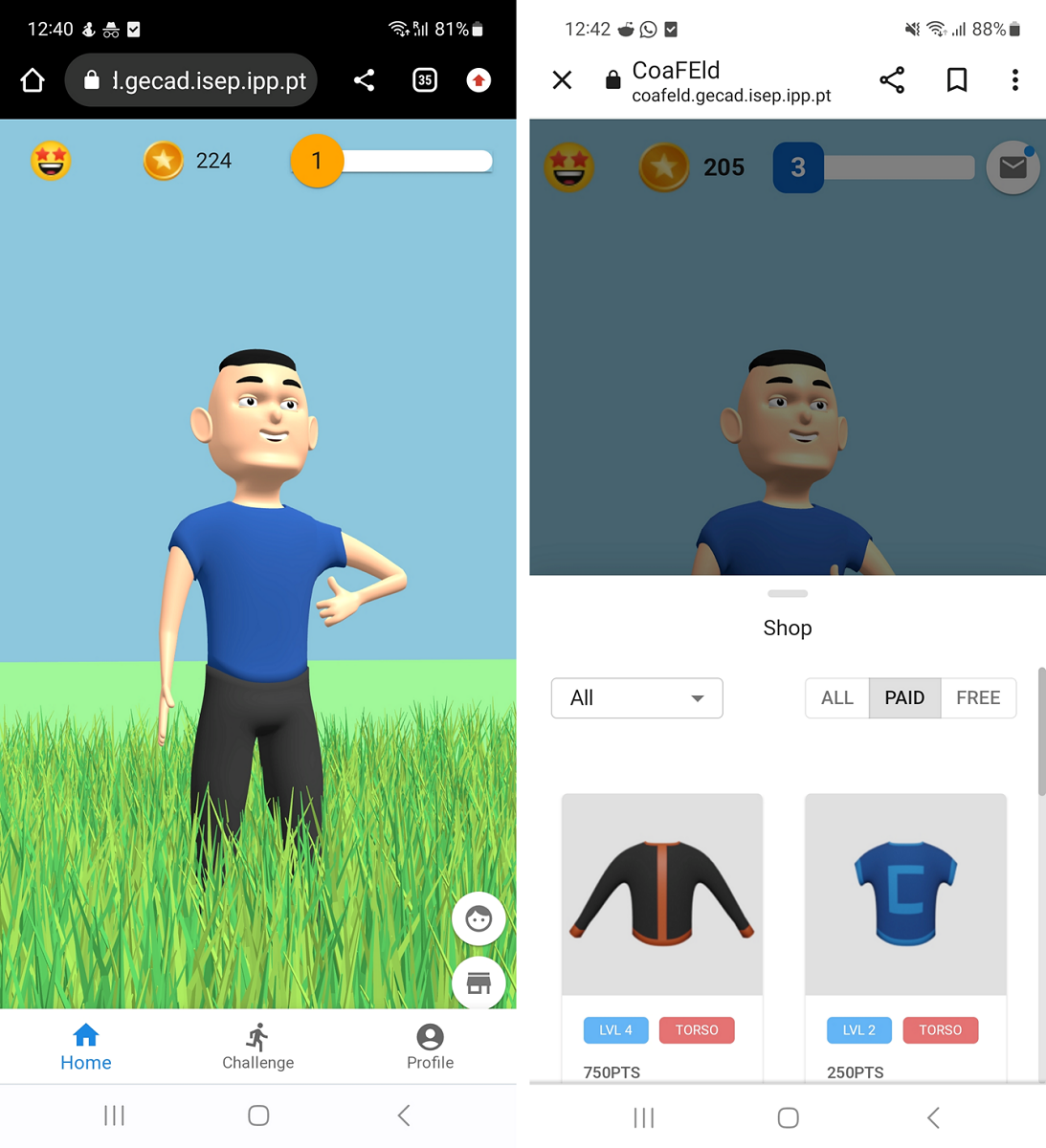
Home interface showing the virtual assistant and gamification components.

One of the most important features available in the application is the home interface with the virtual assistant, which interacts with the user (Figure 3). The application incorporates various gamification techniques. First, the emoji located in the top left corner serves as a visual representation of the user’s emotional feedback (according to the virtual assistant’s current emotional state), indicating positive or negative progress throughout app use. Second, the star coin shows the points earned upon completion of daily challenges, which can be used to exchange and unlock content available in the store, such as clothing items. This feature improves the relationship between the user and the virtual assistant with individual character customization. Third, the blue progress bar, also shown on the screen, represents the user’s earned experience points (XP) and their corresponding level within the application. This visual representation allows users to track their progress and observe their evolution as users of the application.

**Figure 4:**
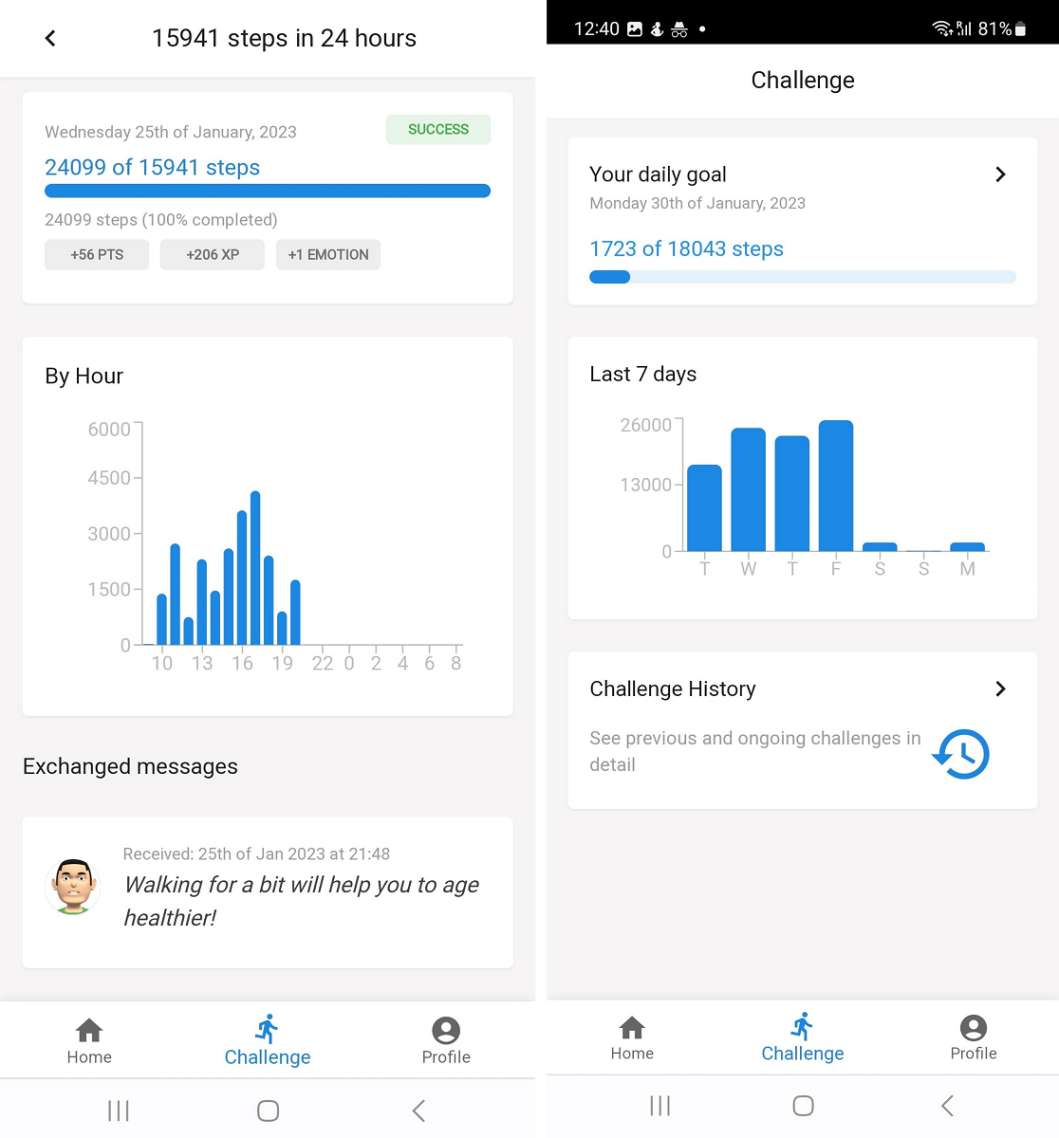
Challenge interface showing the user's daily performance.

The application also includes a Challenge screen (Figure 4) for the user to see their current goals and daily performance, showing the overall progress achieved on each day along with a detailed breakdown of the user’s performance, including aspects such as more and less active hours during the day, points, and experience to be acquired.

**Figure 5:**
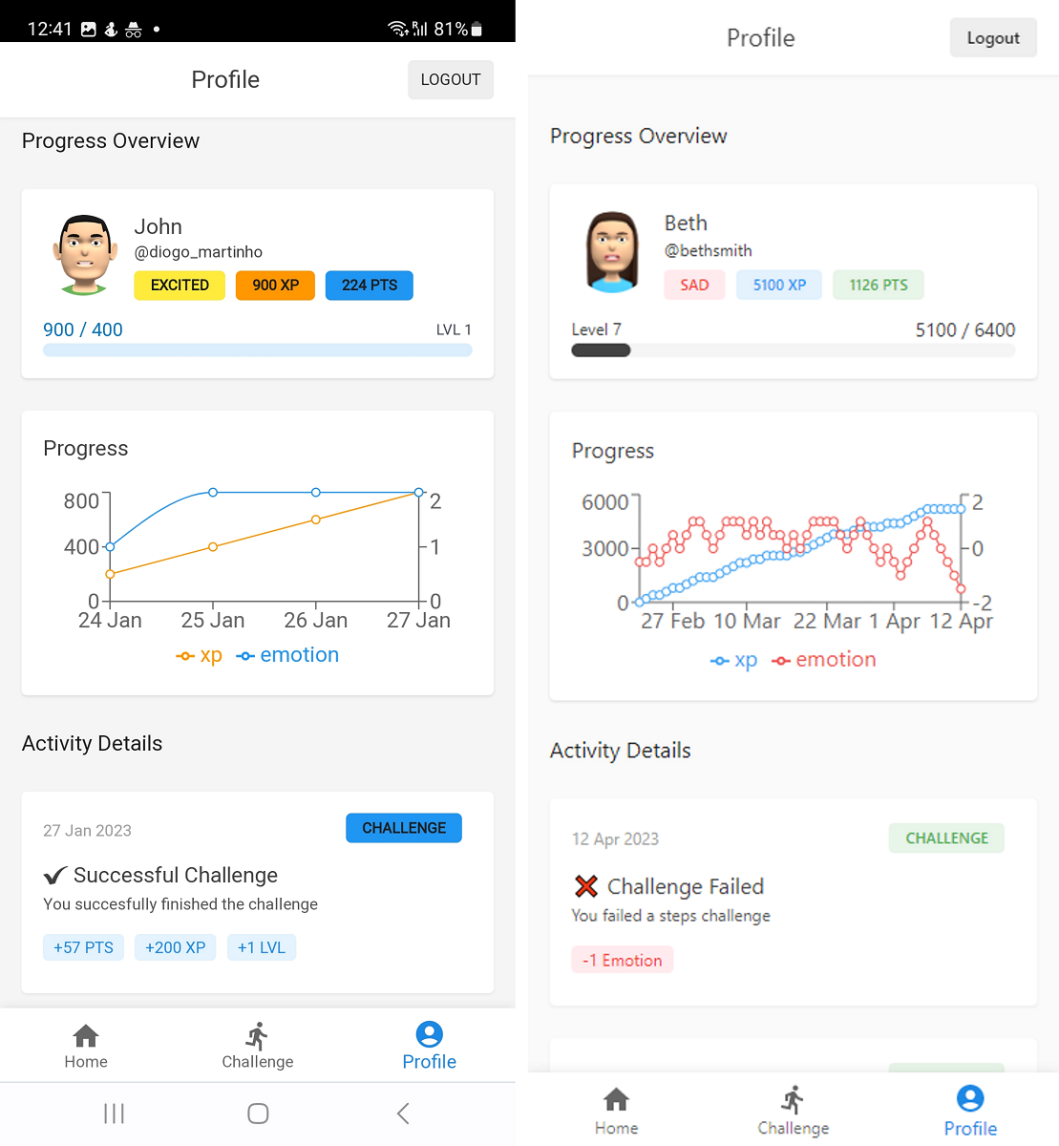
Challenge interface showing the virtual assistant progress.

Finally, in the Profile screen (Figure 5), the user can access the virtual assistant profile, including purchased items, completed challenges, and emotional progress, creating a way to track the user’s progress and achievements.

### Recruitment

The eligibility criteria for participants to enroll in the study were a minimum age of 55 years and an active Google Fit account. We initially recruited 16 participants. However, 3 users dropped out due to smartphone incompatibility with the Google Fit app and 1 due to not having Google Fit data past study enrollment. As a result, the proposed gamified agent-based system was used by 12 participants with a median age of 62 (IQR 12.5) years over 7 days (1 week), and step count data were collected with access to the Google Fit API. Step count data after the intervention were compared with average step count data before the intervention (average daily values over a period of 4 weeks before the intervention). A paired 1-way Student *t* test was used to determine the relationship between the dependent and independent variables. The null hypothesis (*H*_0_≤0) and the alternative hypothesis (*H*_1_>0) were established by comparing the difference between pre- and posttest step count values.

### Questionnaires

After completing the study, each participant was requested to fill out the System Usability Scale (SUS) questionnaire to provide feedback regarding their overall user experience during the period of experimentation.

### Ethical Considerations

No ethics approval was applied for. All participants had access to the data privacy statement with information on data privacy, storage, access, and use within the study, to which they agreed before joining the study. User data were collected according to the permissions each user provided with the use of the Google Fit API. The study was performed according to the privacy policy and policy for the acceptable use of technologies of information and communication of our institution (Polytechnic Institute of Porto). The study was designed as an intervention study comparing pre- and posttest results and was conducted according to the principles of the Declaration of Helsinki and following the General Data Protection Regulation.

## Results

Statistical analysis was performed using an Excel (Microsoft Corp) data analysis toolkit for *t* tests paired with 2 samples for the means method. Step count data were compared for the average of 7 days of experimentation with the average step count data of each corresponding day for the 4 previous weeks (28 days total; average 4 days for each 1 corresponding day).

The step counts were significantly higher after the intervention than before the intervention (mean 4931, SD 5056 steps vs mean 2532, SD 2049 steps and median 3826, IQR 5343 steps vs median 2169, IQR 4888 steps). These data were statistically significant at a 95% confidence level (P=.03; Student *t* test). The measured effect size (Cohen *d*) for paired samples according to 
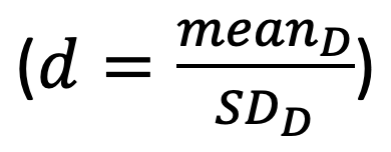
 was 0.65, which is between moderate (effect size >0.5) and large (effect size >0.8).

Looking at average daily differences (Table 1), it is possible to observe a positive difference on all days, with a slight decrease on the third day of the study, which can be explained by the fact that the highest virtual assistant emotional state is achieved after completing 2 daily challenges, starting from the baseline emotional state (0), and on the third day the emotional state no longer improves after successful completion of challenges. Despite this, by establishing a mechanism to reward users with enough points by allowing them to unlock additional content after 5 to 6 days of correct goal completion, it is possible to observe a significant increase in the average daily step count on the last 2 days of the experiment. The last 2 days have the highest average daily difference in step count between pre- and posttest, indicating a positive improvement as the experimentation period continues.

**Table 1:** Average daily difference between step count data before and after the intervention.

Day	Steps, n
1	2572
2	2138
3	975
4	1861
5	1547
6	3031
7	3178

Regarding usability, 8 of the 12 participants completed the SUS questionnaire, which comprises 10 questions, with response options ranging from “strongly agree” to “strongly disagree,” to measure the usability of software, mobile devices, websites, and applications. The answers are then normalized to obtain a usability score to describe the evaluated product. In the case of the developed application, after accessing all the answers provided by the users, we calculated an average usability score of 78 (on a scale of 0-100), corresponding to high usability. Participants were also asked to provide additional comments regarding their experience while using the CoaFEld application, and the overall comments were positive, with only a few suggestions to improve the login interface (since there was no “recover my password” feature implemented and the users were logged off the application automatically after each day).

## Discussion

### Principal Findings

As the world population grows older, it becomes essential to study and develop new personalized health care services that can support older people and promote healthier and active aging. As observed in various studies, different solutions are already being developed that take advantage of intelligent engagement strategies to interact with, persuade, and motivate older people to pursue more positive behaviors in their daily lives. In this context, gamification to enhance traditional health care services, especially when supporting older people, has already been shown to be a strong approach that can provide many advantages that contribute to improved health behaviors.

This study used the CoaFEld application, previously introduced by Martinho et al [30], which was developed with the main goal of supporting older people and offering different functionalities to motivate and persuade them to follow healthier lifestyles. This application includes gamified elements to motivate and enhance user experience, as presented in the design section of this paper. It includes a virtual assistant to interact with the older person and provide feedback on the progress in their health. As was also explained, different emotional states representing the user’s progress are shown, which improve as the user achieves health goals or worsens if they do not.

In our study, we validated the impact of the virtual assistant and the overall gamified solution developed for the CoaFEld application with the participation of users aged 55 years or older. We obtained significant results regarding the approach’s usability, acceptability, and effectiveness. More specifically, our study supports the importance and positive effect of combining gamified elements and a social engagement strategy with a virtual assistant to achieve user progress in a health care setting. The results favor the use of the CoaFEld app, with improved health outcomes after the intervention when compared to the health data obtained before the intervention (P=.02, calculated with the Student *t* test when P<.05) with a Cohen *d* effect size of 0.77, which is moderate yet close to large (ie, effect size >0.8). Additionally, the results of the SUS questionnaire confirmed high acceptability and usability, with an average usability score of 78 along with overall positive comments from the participants, expressing their intentions to keep using the application after the intervention period was completed.

### Limitations

Two main limitations were observed during this validation study. The first limitation relates to the sample size, since 12 participants was an insufficient number to cover all older age groups. As explained in a previous section of this paper, the median age was 62 years, with the oldest participant being aged 70 years. However, as all the users who participated in this study were aged between 55 and 70 years, we can confirm the success of the intervention within this age group. Another limitation was related to the intervention period. For the intervention, participants were asked to use the CoaFEld application for 1 week and, as observed, this allowed us to obtain positive results. However, these results can only confirm a short-term improvement, and a more comprehensive, long-term study is necessary in light of the results obtained in this study.

### Conclusions

Our findings highlight the use and importance of new, intelligent tools and methods to provide personalized support to promote healthier and more active aging among older people. In this context, different engagement strategies have been studied to motivate older people to adopt healthier lifestyles, which include gamifying more traditional health care applications targeted at this age group. These ideas are considered in this study, and a health care service for older people is presented and validated, showing the positive effects of gamification techniques and a virtual cognitive assistant that can enhance the user experience, encourage progress, and provide feedback. Additionally, usability metrics considered for this study confirmed most participants’ high adherence and interest.

Further studies need to be performed on the effectiveness of this approach among older people and to measure the long-term performance of the developed service.

## Appendix

Multimedia Appendix 1 Challenge step count algorithm.

## Abbreviations

API: application programming interface
CoaFEld: Coaching for Elderly
SUS: System Usability Scale
XP: experience points
