# Supplementary material for: Effects of a Gamified Agent-Based System for Personalized Elderly Care: Pilot Usability Study
Source: JMIR Serious Games. 2023 Nov 23;11:e48063. doi: 10.2196/48063 (PMC10704320; doi:10.2196/48063)
Supplement: Multimedia Appendix 1 [file games_v11i1e48063_app1.docx]

| **Algorithm 1. Challenge step count algorithm** |
| --- |
| Input: set S of previous seven days of number of steps, $\left\langle S \right\rangle=7$.  Output: purpose steps value R to the next day.  total_sum ⃪ 0  for step in S:  total_sum += step  end for  average ⃪ total_sum / $\left\langle S \right\rangle$  upperDays ⃪ 0  lowerDays ⃪ 0    for step in S:  if step > average:  upperDays += 1  else if step < average:  lowerDays += 1  end if  end for    if upperDays > lowerDays:  percentage ⃪ 0.05  else if upperDays < lowerDays:  percentage ⃪ -0.1  else:  percentage ⃪ 0  end if    R ⃪ average + average * percentage  return R |
